# Management of myocardial infarction with non-obstructive coronary arteries (MINOCA) in Germany: a single-center study on hospital resources and healthcare economics

**DOI:** 10.3389/fpubh.2024.1407568

**Published:** 2024-10-02

**Authors:** Franz Haertel, Carolin Montag, Thomas Kraeplin, Bernward Lauer, Nedim Memisevic, Sven Moebius-Winkler, P. Christian Schulze, Sylvia Otto

**Affiliations:** ^1^Klinik für Innere Medizin I, Kardiologie, Universitaetsklinikum Jena, Jena, Germany; ^2^Herz - und Gefäßpraxis Gera, Gera, Germany

**Keywords:** MINOCA, hospital resources, ACS, high care monitoring, intensive care, intermediate care

## Abstract

**Background:**

Patients with myocardial infarction with non-obstructive coronary arteries (MINOCA) present as a main feature ≤50% stenosis upon angiography despite clinical symptoms and biomarker elevation related to acute coronary syndrome. Due to broad availability of high sensitivity troponin testing as well as invasive and non-invasive imaging, this clinical entity receives increasing clinical awareness.

**Objective:**

We aimed to investigate the in-hospital work flow and economic impact of MINOCA vs. MICAD (myocardial infarction with obstructive coronary artery disease) patients and related clinical outcomes in a single-center patient collective of a large university heart center in Germany.

**Methods:**

We retrospectively screened and analyzed all patients who were admitted to our hospital under the suspicion of an acute coronary syndrome within a 12-month period (2017–2018) for further diagnostics and treatment. All included patients showed a pathological troponin elevation and received invasive coronary angiography for acute coronary syndrome. Associated in-hospital costs, procedural and various clinical parameters as well as timelines and parameters of work-flow were obtained.

**Results:**

After screening of 3,021 patients, we included 660 patients with acute coronary syndrome. Of those, 118 patients were attributed to the MINOCA-group. 542 patients presented with a “classical” myocardial infarction (MICAD group). MINOCA patients were less frail, more likely female, but showed no relevant difference in age or other selected comorbidities except for fewer cases of diabetes. In-hospital mortality (11% vs. 0%; *p* < 0.001) and 30-day mortality (17.3% vs. 4.2%; *p* < 0.001) after the index event were significantly higher in the “classical” myocardial infarction group (MICAD)- Despite a shorter overall length of hospital stay (9.5 ± 8.7 days vs. 12.3 ± 10.5 days, *p* < 0.01) with a significantly shorter duration of high care monitoring (intensive/intermediate care or chest pain units) (2.4 ± 2.1 days vs. 4.7 ± 3.3 days, *p* < 0.01) MINOCA patients consumed a relevant contingent of hospital resources. Thus, in a 12-months period a total sum of almost 300 days was attributed to high care monitoring for MINOCA patients with a mean difference of approximately 50% compared to patients with classical myocardial infarction. With average and median costs of 50% less per index, MINOCA treatment costs were lower compared to the MICAD group in the hospital reimbursement system of Germany. Consequently, MINOCA treatment was not associated with a relevant profit for these expanses and a relevant share of nearly 40% of the total costs was generated due to high care monitoring.

**Conclusion:**

In light of lower mortality than MICAD and growing scarcity of staff, financial and capacity resources the clinical symptom complex of MINOCA should be put under particular consideration for refining care concepts and resource allocation.

## Introduction

Myocardial infarction with non-obstructive coronary artery disease (MINOCA) is a clinical entity characterized by acute myocardial infarction (AMI) fulfilling the universal AMI criteria (1), the absence of significant coronary artery disease (CAD; stenosis ≥50%) in any coronary artery determined by invasive angiography and no clinically overt specific cause for acute presentation (2).

The prevalence of MINOCA may vary (3). MINOCA has been reported to occur in approximately 6% of patients with AMI (4), other estimates range from a mere 3.5% to a relevant share of up to 25% (2, 5–8) depending on the population being studied and the criteria used to define MINOCA. Consensus statements defining MINOCA by applying specific criteria and diagnostic algorithms have been published in the past years by the European and American cardiac societies (2, 5, 9). However, epicardial and microvascular causes or associated risk factors are overlapping, and establishment of a definite diagnosis can be challenging in clinical practice.

Despite an almost exclusively conservative treatment for MINOCA patients (7, 10–12), this disease entity still utilizes financial and capacity resources including high care monitoring and an in-hospital treatment (13).

At the moment, a health care expenditure discourse is emerging in the western world, particularly in high-income countries such as Germany (14). Recent data clearly show higher than ever growing health care costs *per capita* in Germany that do not translate to longevity advantages (14). On the contrary, an economically strong country like Germany can be viewed as a “poor performer” compared to other nations regarding health care outcomes (14). This discrepancy between rising health care costs *per capita* and no added benefit or even a decrease in life expectancy in Germany is creating a growing awareness for a more cautious resource allocation (14).

In light of this context, data on in-hospital resource management and economic aspects for the clinical entity of MINOCA are scarce and new insights are strongly needed.

## Aim of the study

The present study aimed to investigates clinical characteristics, prognosis, in-hospital workflow, and consequently economic impact of MINOCA patients compared to “classical” AMI patients [myocardial infarction with obstructive coronary artery disease (MICAD)], and analyses potential implications for hospital resource management of MINOCA.

## Materials and methods

### Study design

At the University Hospital Jena, Germany, a “MINOCA database” was established in April 2017 as the foundation for the data used in this study. The study received approval from the medical faculty’s ethics commission at the Friedrich-Schiller University Jena (registry number: 2018-11-35-Daten). This study is a single-center, retrospective study that analyzed data from a one-year period (April 2017 to May 2018) and was conducted in accordance with the Declaration of Helsinki.

### Patient population

The study screened in a first step all patients who received invasive coronary diagnostics for a working diagnosis of acute coronary syndrome (ACS), including ST-elevation myocardial infarction (STEMI) and non-ST elevation myocardial infarction (NSTEMI) as well as elevated troponin levels with no or indistinct signs of myocardial ischemia. From this population, patients were selected further for suspected MINOCA. Cases were assessed using a specific diagnostic algorithm designed to distinguish MINOCA from other causes involving non-ischemic mechanisms in line with a previous study (9).

Patients who received a coronary angiography for other indications such as elective or preoperative diagnostics or who had normal cTn levels were excluded from the study.

We used the “Universal definition of myocardial infarction (UDMI)” from 2018 for elevation of cTn levels to define AMI (1): “detection of an elevated cTn value above the 99th percentile upper reference level (URL) is defined as myocardial injury.” For an acute injury, cTn values had to elevated and/or rise. Additionally, a clinical constellation compatible with acute myocardial injury (typical ischemic symptoms, new ischemic electrocardiogram (ECG) – changes or new pathological Q-waves, imaging evidence of loss of viable myocardium or new regional wall abnormalities, or detection of intracoronary thrombus) was necessary. For evaluation of the clinical constellation, the working diagnosis of in-hospital course was considered and reevaluated in the aforementioned process.

### Definition of MINOCA diagnosis

Definition of MINOCA was based on current consensus and guidelines (2, 9, 15). First, the working diagnosis of MINOCA was established on the initial presentation of the patient and all relevant information using the hospital information system upon coronary angiography, if the following criteria were fulfilled: universal AMI criteria, non-obstructive coronary arteries <50% in any potentially infarct-related artery and no clinically overt specific cause for the acute presentation during initial work-up and upon decision for coronary angiography.

All included cases were retrospectively reviewed and reanalyzed regarding the final diagnosis of MICAD or MINOCA using all available information from the hospital information system (e.g., laboratory values, imaging results, angiographic results). In case of incongruent findings, final diagnosis was established by clinical consensus reading by two investigators (C.M. and S.O.).

Due to the retrospective nature of this study, patients with a specific alternative diagnosis such as pulmonary embolism, sepsis, Tako-Tsubo syndrome, myocarditis or other non-cardiac cTn elevation [e.g., stroke, acute respiratory distress syndrome (ARDS)] were excluded.

In addition, radiological findings were analyzed to determine if and which imaging was performed (cardiac magnetic resonance imaging (MRI), coronary computed tomography (CT) angiography, myocardial scintigraphy).

### Troponin test

cTn testing was conducted utilizing the Cobas e 801^©^ module, a component of the Roche Cobas 8,000^©^ system by Roche^©^ employing the ElectroChemiLuminescence Immunoassay (ECLIA) methodology. The reference values at our study site for normal troponin levels were established as <9 pg./mL for women and < 16.8 pg./mL for men. Notably, concentrations of cTn exceeding the 99th centile of the URL were considered elevated as mentioned above.

### Data management

Various demographic, clinical and procedural parameters, as well as laboratory parameters and diagnostic work-up were collected and anonymously registered using the SAP^®^ electronic patient management system (SAP^®^, Walldorf, Germany). Also, emergency medical protocols (obtained from emergency medical services (EMS) and the emergency department) were used for confirming the initial working diagnosis MINOCA. We determined in-hospital and 30-day mortality as both all-cause and cardiovascular mortality.

The electronic documentation system of the cardiac catheterization laboratory, cardWorks^®^ (Schwarzer Cardiotek GmbH, Heilbronn, Germany), and coronary angiography findings were reviewed and provided specific clinical parameters such as ejection fraction, heart rate, and blood pressure, as well as information on the interventions performed, percutaneous coronary intervention (PCI), and intracoronary imaging [optical coherence tomography (OCT) or intravascular ultrasound (IVUS)]. The degree of stenosis in diagnosed CAD (exclusion, mild CAD with stenosis <50%; manifest CAD with stenosis ≥50%) were determined.

### In-hospital workflow data

We collected logistical data that were obtained from specific time points that every patient underwent within the hospital from first presentation to diagnostics and to discharge. Relevant parameters and measurements that we investigated were: primary point of contact with the hospital (e.g., emergency department), rate of admission to high-care monitoring, time interval between admission to the hospital and arrival in the catheterization laboratory, length of in-hospital stay, length of high care monitoring, mode and time of admission to the hospital and seasonal distribution of admission among others.

### Economic data

Costs associated with the studied patients were derived from the German health care insurance reimbursement system. In Germany, hospital costs/health care services are classified and reimbursed through the so-called Diagnosis Related Groups (DRG) system. It operates on a case-based payment model, assigning a fixed reimbursement amount for each patient case, considering factors like diagnosis, procedures, age, and gender. This system aims to streamline billing processes, enhancing transparency and efficiency in healthcare services.

Key features of the German DRG system include:

Case-Based Payments: Hospitals receive a predetermined reimbursement for each case, covering all associated costs related to a specific condition or procedure.
Patient Classification: Patients are categorized into specific DRGs based on diagnoses and procedures, with each DRG having an associated base rate that is adjusted based on factors such as age and comorbidities.
Grouping Criteria: The classification of patients into DRGs relies on standardized criteria, including primary and secondary diagnoses, procedures, age, and other relevant factors.
Coding System: Accurate coding of diagnoses and procedures using the international classification of diseases (ICD) and operational and procedural system (OPS) coding systems is crucial for correct DRG assignment.
Transparent Reimbursement: The DRG system provides transparency by specifying in advance the reimbursement amount for each case, enabling hospitals to make informed decisions about resource allocation and budgeting.
Updates and Adjustments: Periodic updates ensure the DRG system remains current, reflecting changes in medical practices, technology, and healthcare policies.

To characterize the economic impact of MINOCA, the costs (in EUR) were categorized as follows: (1) procedures, which included invasive angiography and additional intravascular imaging if necessary, (2) high-care monitoring such as intensive care unit (ICU), intermediate care unit (IMC), and chest pain unit (CPU), including costs for medical supplies, care, medication, ventilation, dialysis, and extracorporeal circulatory support, (3) regular ward, which included costs for medical supplies, care, and medication, and (4) total cost.

### Statistical analysis

Analyses were done using SPSS Statistics (version 27.0, SPSS Inc., IBM, Armonk, New York). A *p*-value less than 0.05 was considered statistically significant, a *p*-value <0.1 was considered a trend. Baseline parameters are presented descriptively including whole integers and percentages. The data was analyzed by Kolmogorov-Smirnov test for normal distribution and the appropriate statistical tests were applied. The frequency of nominally scaled parameters was compared using Pearson’s chi-squared test. Variables are expressed as mean ± standard deviation (Mean ± SD) and the *t*-test for independent samples and one-way analysis of variance were used for mean comparisons. Non-normally distributed data were expressed as median with interquartile range (Median [IQR: 25th percentile–75th percentile]) and compared using the Mann-Whitney U-test. The diagrams depicted were generated using SPSS Statistics.

## Results

### Study population and clinical characteristics

**Figure 1:**
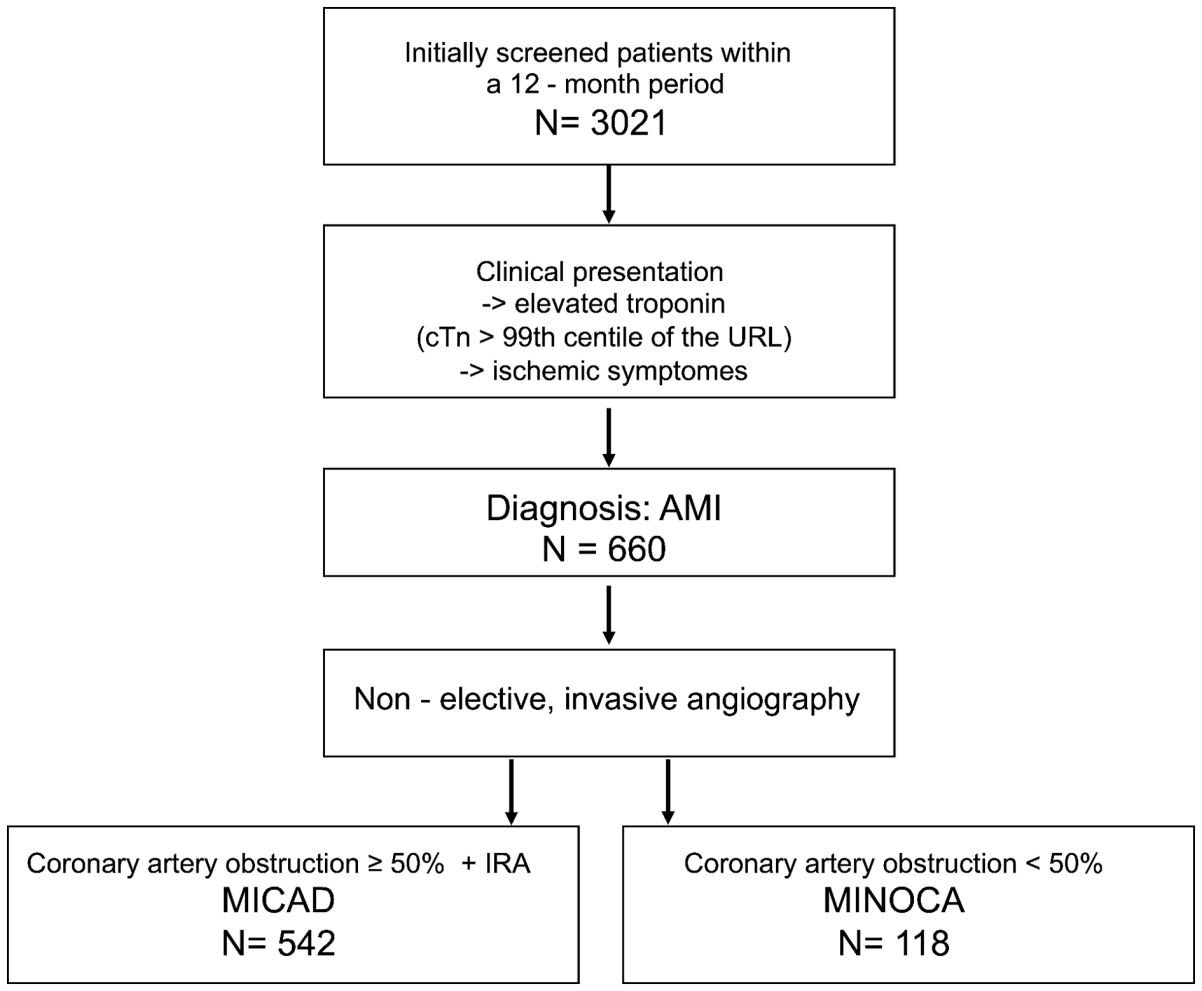
Flow chart of the patient selection for this study. MINOCA, myocardial infarction with non-obstructive coronary arteries; MICAD, myocardial infarction with obstructive coronary artery disease [ST elevation myocardial infarction (STEMI) and non-ST elevation myocardial infarction (NSTEMI)]; N, absolute number; cTn, cardiac troponin; URL, upper reference level; AMI, acute myocardial infarction; IRA, infarct related artery.

In the time frame of 12 months, 3,021 patients were screened. A total of 660 patients were included in the study. Of those, 542 patients (82%) were attributed to the MICAD group and 118 patients (18%) formed the MINOCA group (Figure 1). Table 1 summarizes the baseline characteristics for the entire study population. The prevalence of MINOCA was 18% in our study population of patients presenting with ACS symptoms.

**Table 1:** Baseline characteristics.

	Total study population	MICAD	MINOCA	*p*-value*
	(*n* = 660)	[*n* = 542 (82%)]	[*n* = 118 (18%)]	
**Demographics**				
Age [years – mean ± SD]	68.8 ± 12.8	68.8 ± 12.8	69.8 ± 12.7	n.s.
Male [*n* (%)]	413 (62.6)	365 (67.3)	48 (40.7)	<0.001
Female [*n* (%)]	247 (37.4)	177 (32.7)	70 (59.3)	<0.001
BMI [kg/m^2^ – mean ± SD]	28.3 ± 5.2	28.4 ± 5.1	27.5 ± 5.4	n.s.
Charlson comorbidity index [mean ± SD]	4.78 ± 1.8	4.87 ± 1.8	3.9 ± 1.7	<0.001
**All-cause mortality**				
In-hospital [*n* (%)]	59 (9)	59 (11)	0 (0)	<0.001
30-day [*n* (%)]	99 (15)	94 (17.3)	5 (4.2)	<0.001
**Cardiovascular mortality**				
In-hospital [*n* (%)]	39 (5.9)	39 (7.2)	0 (0)	<0.001
30-day [*n* (%)]	58 (8.8)	54 (9.9)	4 (3.3)	<0.001
**Echocardiographic parameters**				
LVEF [% – mean ± SD]	49.5 ± 15.2	41.8 ± 14.9	56.1 ± 15.6	<0.001
**Hemodynamic parameters**				
Systolic blood pressure [mmHg – mean ± SD]	141.5 ± 36.1	139.6 ± 35.4	151.9 ± 38.1	<0.001
Heart rate [bpm – mean ± SD]	79.5 ± 22.4	79.7 ± 22.3	78.7 ± 23.1	n.s.
**Biomarkers**				
Troponin [pg/ml–median [IGR]]	4165.1 [569.2–26519.3]	6761.6 [729.5–35908.5]	821.1 [197.1–3642.4]	<0.001
CK [pg/ml–median [IGR]]	4.8 [2.4–15.4]	5.8 [2.7–18.6]	2.5 [1.8–4.7]	<0.001
Creatinine [μmol/l – mean ± SD]	115.2 ± 88.4	115.9 ± 89.3	111.5 ± 83.7	n.s.
**Organizational work-flow and resources**				
Admission via ED [*n* (%)]	515 (78)	431 (79.5)	84 (71.2)	n.s.
High-care monitoring [*n* (%)]	560 (84.8)	487 (89.9)	73 (61.9)	<0.01
CABG [*n* (%)]	51 (7.7)	50 (9.2)	0 (0)	< 0.01
Interval admission ED-catheterization laboratory [h–median [IQR]]	11.3 [1.5–65.5]	9.1 [1.1–59.9]	43.1 [10.4–117.6]	<0.001
**Time of admission**				
Morning [*n* (%)]	204 (30.9)	184 (33.9)	29 (24.5)	n.s.
Afternoon [*n* (%)]	314 (47.6)	267 (49.2)	64 (54.1)	n.s.
Night [*n* (%)]	110 (16.7)	91 (16.8)	25 (21.4)	n.s.
On-hours [*n* (%)]	404 (61.2)	333 (61.4)	71 (60.4)	n.s.
Off-hours [*n* (%)]	256 (38.8)	209 (38.6)	47 (39.6)	n.s.
Weekend [*n* (%)]	117 (17)	107 (19.7)	10 (8.5)	0.01
**Comorbidities**				
Hypertension [*n* (%)]	497 (75.3)	419 (77.3)	78 (66.1)	n.s.
Heart failure [*n* (%)]	277 (42)	232 (42.8)	45 (38.1)	n.s.
PAD [*n* (%)]	46 (7.0)	40 (7.4)	6 (5.1)	n.s.
Stroke [*n* (%)]	23 (3.5)	22 (4.1)	1 (0.8)	n.s.
Dementia [*n* (%)]	15 (2.5)	13 (2.4)	2 (1.7)	n.s.
COPD [*n* (%)]	45 (6.8)	35 (6.4)	13 (11.2)	n.s.
Diabetes [*n* (%)]	226 (34.2)	287 (36)	27 (22.9)	0.037

*p* < 0.05 = statistically significant; * MICAD vs. MINOCA; MICAD, myocardial infarction with obstructive coronary artery disease; MINOCA, myocardial infarction with non-obstructive coronary arteries; ED, emergency department; SD, standard deviation, BMI, body mass index; ICU, intensive care unit; CABG; coronary artery bypass graft; h, hours; PAD, peripheral artery disease; COPD, chronic obstructive pulmonary disease; CAD, coronary artery disease; n.s., not significant; IQR, Interquartile range; CK, creatinine kinase.

The total study population included 62.6% men (413 patients) and 37.4% women (247 patients). The age at presentation ranged from 25 to 96 years, with a mean of 68.8 ± 12.8 and a median of 72 [60–79] years. MINOCA patients were more likely female (59.3% vs. 32.7% *p* < 0.001), but showed no difference in age compared to the MICAD group.

A better left ventricular ejection fraction (56.1 ± 15.6% vs. 41.8 ± 14.9%; *p* < 0.001) and more hypertensive blood pressure values (151.9 ± 38.1 mmHg vs. 139.6 ± 35.4 mmHg; *p* < 0.001) prior to the angiography were found in MINOCA patients.

In general, the presence of diabetes mellitus, hypertension or multimorbidity seem to be important co-factors for the occurrence of cardiovascular diseases in both groups (Table 1).

### Biomarkers

Upon presentation, MINOCA patients showed lower cardiac biomarkers (troponin: 821.1 pg. / ml [197.1–3642.4] vs. 6761.6 pg. / ml [729.5–35908.5]; *p* < 0.001; CK: 2.5 pg. / ml [1.8–4.7] vs. 5.8 pg./mL [2.7–18.6]; *p* < 0.001) (Table 1). All but two patients in the MINOCA-cohort, and all patients in the MICAD-cohort had a clear troponin elevation >5 x URL.

There was no statistically significant difference in the minimal hemoglobin levels between the two groups (7.03 ± 1.48 mmoL/L vs. 6.79 ± 1.63 mmoL/L; *p* < 0.238). The leukocyte count was found to be significantly higher in the MICAD group compared to the MINOCA group (14.29 ± 6.67 Gpt/l vs. 12.83 ± 13.68 Gpt/l; *p* < 0.001). Moreover, maximal CRP levels were significantly higher in the MICAD group compared to the MINOCA group (84.70 ± 106.18 mg/L vs. 66.24 ± 91.99 mg/L; *p* < 0.004). Although there was no statistically significant difference in the GFR (65.64 ± 24.68 mL/min vs. 67.26 ± 26.74 mL/min; *p* = 0.482) and serum creatinine levels (106.3 ± 83.45 μmoL/L vs. 110.88 ± 84.21 μmoL/L; *p* = 0.173) between the two groups at admission, the serum creatinine levels in the MICAD group were significantly higher than in the MINOCA group during the follow-up period (93.92 ± 68.81 μmoL/L vs. 109.62 ± 86.56 μmoL/L; *p* = 0.003) (Table 1).

### Mortality and outcomes

The total study population had a 30-day all-cause mortality rate of 15% (99 patients). Compared to the MINOCA group, the MICAD group had significantly higher rates of all-cause in-hospital mortality (11% vs. 0%, *p* < 0.001) and all-cause 30-day mortality (17.3% vs. 4.2%, *p* < 0.001) following the clinical index event (Table 1). The majority of both in-hospital and 30-day mortality cases were attributed to cardiovascular causes.

### Organizational work-flow and in-hospital resources

**Figure 2:**
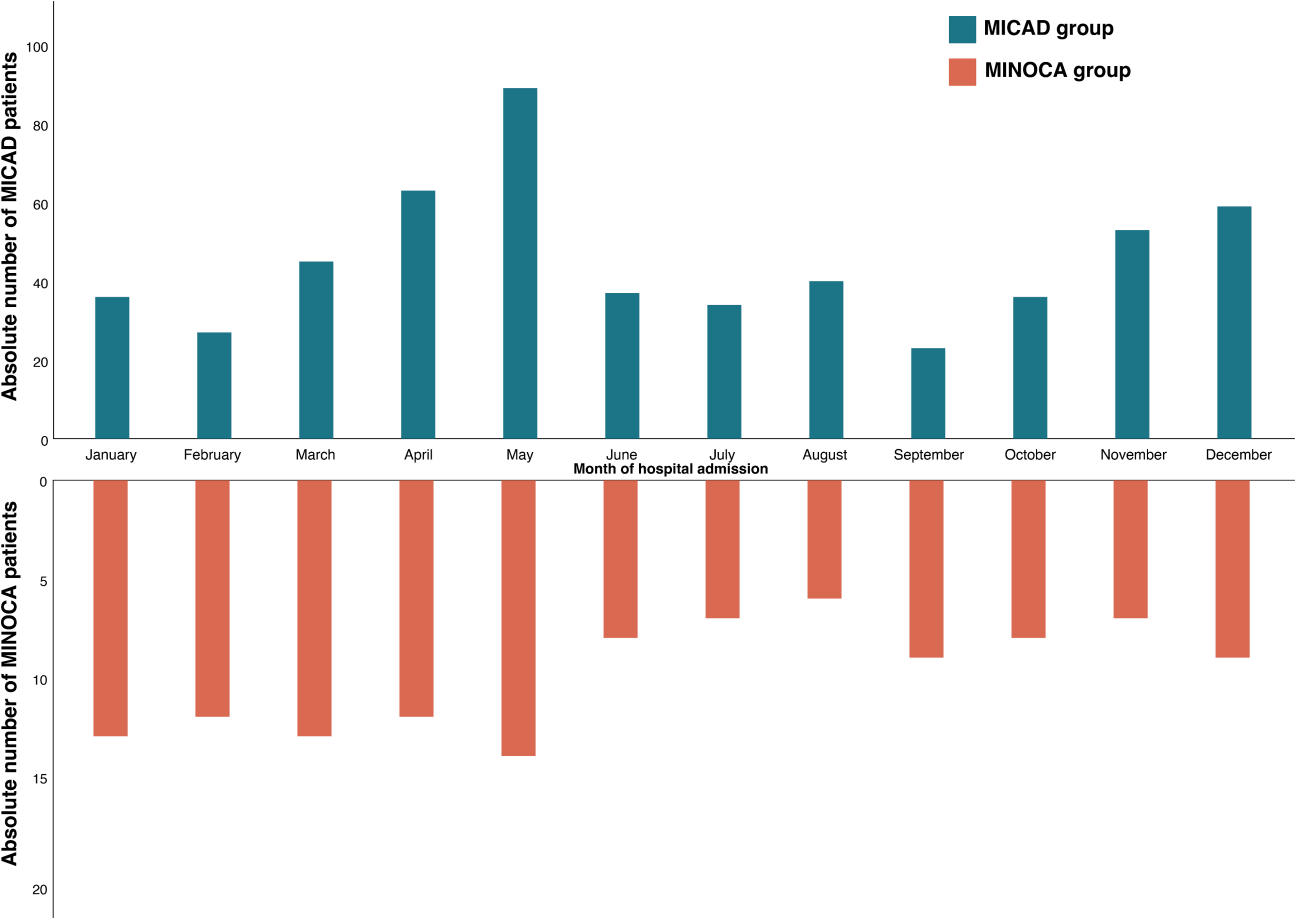
Seasonal distribution of the study population. Comparison of MINOCA (orange) vs. MICAD (blue); MINOCA, myocardial infarction with non-obstructive coronary arteries. MICAD, myocardial infarction with obstructive coronary artery disease [ST elevation myocardial infarction (STEMI) and non-ST elevation myocardial infarction (NSTEMI)].

The emergency department was the primary point and most common mode of hospital admission for >70% of the entire patient cohort with no difference between MICAD and MINOCA patients (Table 1). Approximately 50% of the patients arrived in the afternoon but still within regular working hours on a weekday. MINOCA patients were less present during the weekend [compared to MICAD patients (8.5% vs. 19.7%; *p* = 0.01, Table 1)]. With regard to potential seasonal fluctuations of these entities, both MINOCA and MICAD cases showed a similar pattern with two peaks in late spring and late fall (Figure 2).

MINOCA patients were more likely to receive coronary angiography later after admission (43.1 h [10.4–117.6] vs. 9.1 h [1.1–59.9]; *p* < 0.001) (Table 1).

Overall, both MINOCA and MICAD required relevant, approximately one-week long in-hospital treatment. However, length-of-hospital stay (mean: 9.5 ± 8.7 days vs. 12.5 ± 10.5 days; *p* < 0.01; median: 8.0 days (5–14) vs. 7.0 days (4–11); *p* = 0.023) was significantly shorter in the MINOCA group compared to MICADs.

**Figure 3:**
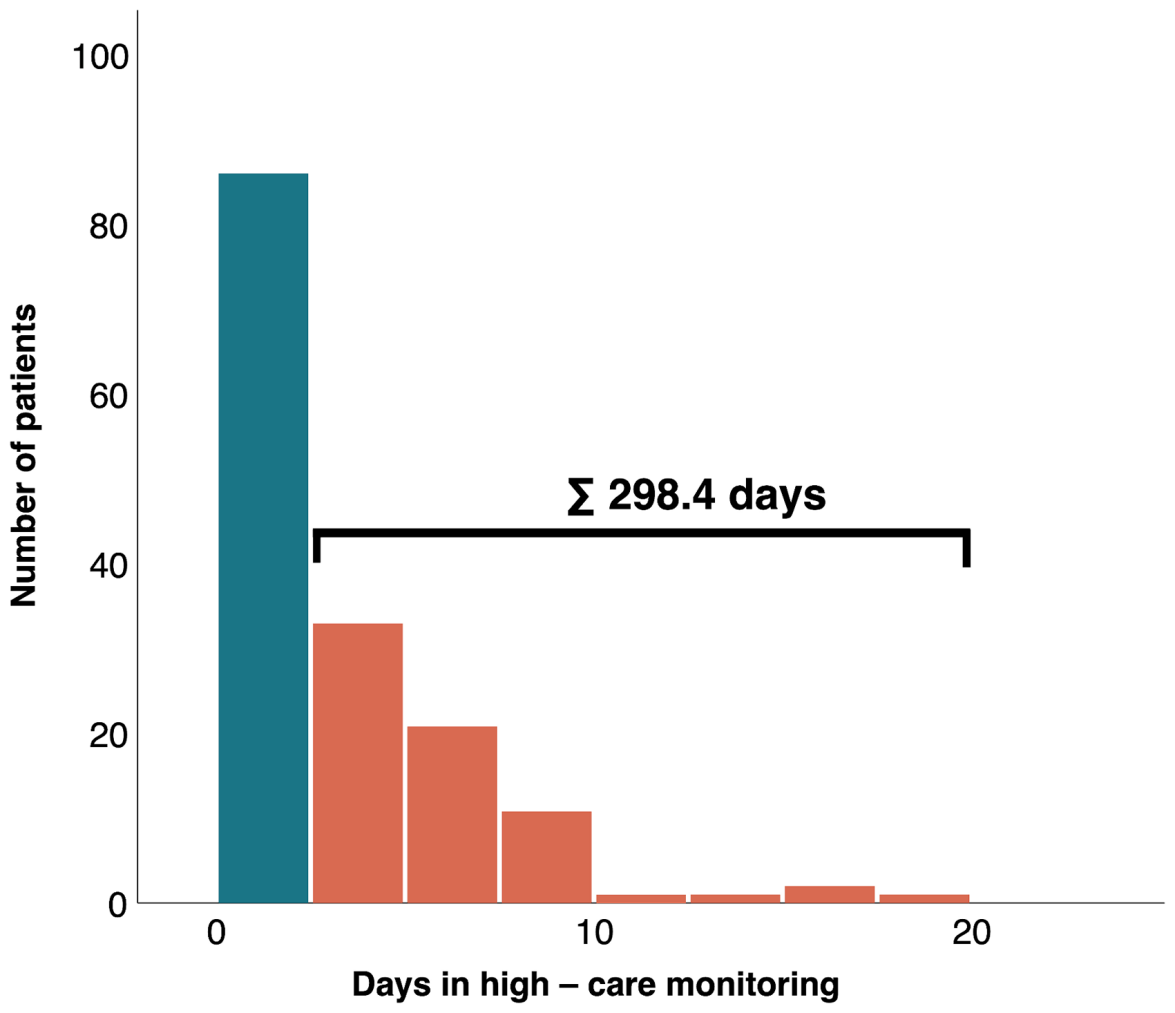
Absolute number of days of MINOCA patients in high care monitoring (orange) and without high-care monitoring (blue). MINOCA, myocardial infarction with non-obstructive coronary arteries.

Demands for high care monitoring (ICU, IMC, CPU), showed significant differences for admission rates (61.9% vs. 89.9%; *p* < 0.01) and length of stay in these units (mean: 2.4 ± 2.1 days vs. 4.7 ± 3.3 days; *p* < 0.01; median: 1.9 days [1.1–4.4] vs. 1.5 days [0.4–2.7]; *p* = 0.01) between MINOCA and MICAD patients. Nevertheless, a total sum of 298.4 days was utilized solely by MINOCA patients for high care monitoring in a 12-months period (Figure 3).

### Analyses of in-hospital costs and reimbursement

With average and median costs of 6871.5 ± 5670.8 EUR and 3585.2 [3461.4–10167.2] EUR per index, MINOCA treatment costs were lower compared to the MICAD group (mean: 13045.9 ± 7896.9 EUR; *p* = 0.02; median: 5958.9 [4682.7–16654.9] EUR; *p* < 0.01) with a mean difference of approximately 6,000–7,000 EUR. However, MINOCA treatment was not associated with a relevant profit for these expanses (mean: 198.1 ± 4329.2 EUR//median: 0 (−1702.6–1405.1) EUR) in the German health care system.

**Figure 4:**
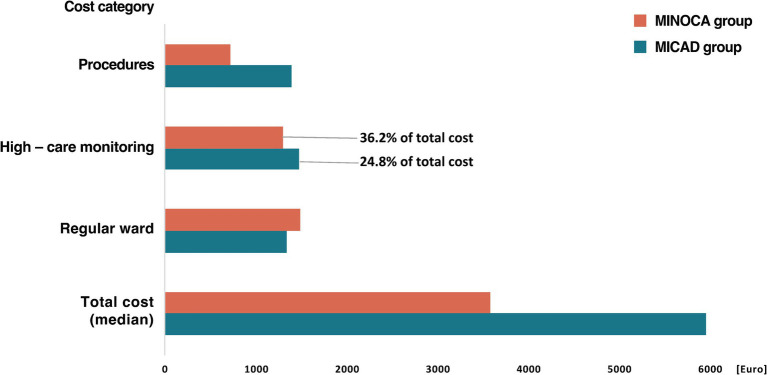
Median total hospital cost per index for MICAD and MINOCA patients and the respective cost category in group comparison. Definition: Procedures = invasive angiography including additional intravascular imaging if necessary. High-care monitoring = intensive care unit (ICU), intermediate care unit (IMC), and chest pain unit (CPU) including costs for medical supplies, care, medication, ventilation, dialysis, and extracorporeal circulatory support. Regular ward = regular admission including costs for medical supplies, care, and medication. MINOCA, myocardial infarction with non-obstructive coronary arteries; MICAD, myocardial infarction with obstructive coronary artery disease.

For MINOCA patients, 36.2% of the total costs were attributed to high care monitoring and 41.5% to regular ward care, in comparison, these expenditures for MICAD patients were 24.8 and 22.6%, respectively (Figure 4).

## Discussion

The aim of this study was to investigate the procedural and economic impact of MINOCA patients compared to those with “true myocardial” infarction and related clinical outcomes in a single-center patient collective of a large university heart center in Germany. Our results showed that MINOCA patients had lower cardiac biomarkers, a better and mostly preserved left ventricular ejection fraction, and showed no differences in age, very low in-hospital and 30-day mortality.

Although MINOCA patients had shorter hospital stays and spent less time in high care units, they still required significant in-hospital resources over a 12-month period. Treatment costs were lower for MINOCA patients but did not result in significant profit from the German diagnosis-related group (DRG) system. These findings underscore the importance of distinguishing between MINOCA and MICAD in patient management and resource allocation, especially in the context of limited resources.

### Classification and prevalence of MINOCA

The diagnosis of MINOCA was made based on the initial presentation of the patient together with criteria adapted from Agewall et al. (2) from European Society of Cardiology (ESC) working group position paper on myocardial infarction with non-obstructive coronary arteries.

Recently, there has been further refinement of terminology based on pathophysiology of MINOCA. The term *MINOCA* should be reserved for patients with evidence of ischemia-related myocardial necrosis, and thus is suggestive for an epicardial cause of AMI such as coronary dissection, coronary artery spasm or coronary plaque (rupture or erosion). Whereas the novel term *TpNOCA* (Troponin-positive with non-obstructive coronary arteries) was formed and refers to normal or regional wall abnormalities with a microvascular pattern (e.g., Tako-Tsubo-Syndrome, myocarditis, coronary microvascular spasm or coronary embolism) (6).

Per definition MINOCA is used as a “working diagnosis” and should not be considered after a specific cardiac (coronary or non-coronary) or extra-cardiac condition has been identified. However, current consensus (6, 15, 16) also utilizes “unclassified MINOCA” (= MINOCA-working diagnosis vs. unclassified MINOCA) as a specific cardiac condition after additional investigation such as vascular function tests. Non-invasive and intracoronary imaging, as well as extra-cardiac work-up. After this diagnostic process, an alternative diagnosis (e.g., sepsis, pulmonary embolism, cardiac contusion) or cardiac/coronary diagnosis (e.g., Tako-Tsubo syndrome, myocarditis, coronary dissection or thrombus) can be established.

However, since our work is a retrospective analysis, patients correctly attributed to the MINOCA group remained MINOCA patients but the final, specific diagnosis (if established) was determined and documented in our database. One could argue, if an alternative diagnosis such as sepsis or pulmonary embolism should even be considered as a MINOCA working diagnoses or if the clinical context was misjudged or not overtly present during the initial patient assessment. Furthermore, MINOCA or TpNOCA terminology should solely be attributed to epicardial or myocardial pathophysiological causes of myocardial injury. This however, is an ongoing debate (6, 15–18). Despite its many advantages, it must also be said that the use of hs-cTnT test themselves might “produce” the diagnosis of MINOCA as the reliability of positive values is challenged by noncoronary causes and thus contributes to over-diagnosis and over-investigation (19, 20).

With a share of 18%, MINOCA patients make up a significant proportion of all myocardial infarctions in the analyzed patient population. Regarding the prevalence of MINOCA, there have been many discrepancies and no clear statements in recent years due to the lack of a uniform definition: The term “MINCA” was already described in 2000, encompassing myocardial infarctions in patients without atherosclerosis of the epicardial vessels (21). According to the study conducted at that time, these patients had a lower cardiovascular risk profile and an “excellent” prognosis (21). The term MINCA was replaced by MINOCA by John Beltrame et al. (22, 23), so that patients with angiographic stenosis between 1 and 50% were also included (23).

The position paper of the ESC states the prevalence of MINOCA to be 1–13% of all patients with acute myocardial infarction (2) and a systematic review by Pasupathy et al. (4) based on 28 publications reveals a prevalence of approximately 6% of patients presenting with ACS symptoms. A prospective multicenter large-scale cohort study by Lawless et al. (24) with 13.202 patients determined a 10.9% prevalence of MINOCA patients in this collective and two very recent studies, one prospective by Bergamaschi et al. (5) and one retrospective meta-analysis by Mileva et al. (25) reported 6 and 22%, respectively.

Our own study findings corroborate a comparable percentage of 17.9% within an ACS collective despite its retrospective nature and excluding diagnosis like pulmonary embolism or sepsis. This might be due to improvement of early screening methods, the use of high-sensitive troponin assays, and possibly a higher awareness of MINOCA as an ACS entity (26).

Intracoronary imaging such as IVUS or OCT was performed in only 16 patients (2.4%) of the entire study cohort, which is rather low. However, we believe it reflects the true real-life situation and might be a bit higher today (5 years forward) due to the increasing evidence and clear guideline recommendations. The strength of our work is the angiography that was carried out for all patients and is not required in all ACS patients regarding current guidelines. Therefore, one could speculate that in an ACS cohort of patients without 100% invasive diagnostic the prevalence of MINOCA might be even higher.

### Comorbidities, high-sensitivity troponin assays and mortality

The gender distribution in this study is in slight favor for women (59.3%). With a median of 74 years [60–79] and average age of 69.8 ± 12.7 years, there were no significant differences in terms of age between the two groups. This is consistent with the work published in 2022 by published in 2022 by Lopez-Pais et al. (26). Here, an average age of 64.6 ± 14.9 years or 66.7 ± 13.5 years was described (26). In the VIRGO study, MINOCA was more often found in younger patients; these patients were more likely to have an NSTEMI and fewer traditional cardiac risk factors than patients with MICAD (27). Contrary to the VIRGO study, the MINOCA patients in the described collective were not significantly younger, which contradicts the assumption that MINOCA is supposedly a disease of younger people.

Comorbidities and cardiovascular risk factors were evenly distributed in the study cohort; and without differences between MICAD and MINOCA patients with the exception of a preexisting type 2 diabetes, which was more often seen in the MICAD group.

Troponin is considered an important prognostic marker (15). MINOCA patients have significantly lower concentrations of cardiac necrosis markers as opposed to classical myocardial infarction where the massive release of hs-cTnT (high sensitive cardiac troponin T) occurs as a result of significant damage to the supplied myocardial area due to the immediate total or subtotal occlusion of a coronary artery (15, 28). Moreover, an acute troponin elevation of >5 x URL has a very high positive predictive value for myocardial ischemia compared to troponin elevation ≤3 x URL (8, 9, 29).

Since this is not the case in MINOCA, it can be assumed that there is a lower release of troponin.

This could partially explain why MINOCA patients had a lower mortality rate. Although the MINOCA group had no in-hospital deaths, their 30-day mortality rate of 4.2% was significantly lower than the 17.3% observed in the classic myocardial infarction group. However, this mortality rate should still be regarded as substantial and clinically relevant. The study by Lawless et al. (24) mentioned earlier, which compared female and male MINOCA patients, reported an in-hospital mortality of 2.1 to 3.2%; a 1-year mortality of 6.9 to 9.2% and long-term mortality 11.2 to 14.2%. A more recent study by Bergamaschi et al. (5) reports a mean mortality rate of 8.1% (over a period of 33.7 ± 12.0 months).

Left ventricular ejection fraction at admission was better in the MINOCA group, which could potentially speak for a better long-term prognosis for MINOCA patients. However, it is extremely difficult to make a specific statement about long-term prognosis due to the large heterogeneity of available registries. For example, Lopez-Pais et al. (26) describe similar complications such as reinfarction, severe bleeding, stroke, pulmonary edema, or shock in 13.8% of MINOCA patients vs. 17.6% in MICAD (*p* = 0.335).

### Economic aspects and in-hospital resource management

While the DRG system promotes standardization and transparency, critics argue that it may incentivize hospitals to increase case volume to maximize revenue. Concerns also exist about potential underpayment for complex cases requiring more resources.

In 2020, healthcare expenditure in Germany amounted to 13.1% of GDP (Federal Statistical Office of Germany). Ischemic heart diseases and acute myocardial infarction accounted for 10.8 billion Euros (73.8% attributed to ischemic heart, 26.2% acute myocardial infarction), which is a 15.3% increase from 2015. As the population ages and risk factors increase, these costs are likely to continue to rise. It is worth mentioning that Germany has already the highest *per capita* expenditures annually for cardiovascular diseases and with more than 900 Euros per person, Germany is also the unchallenged leader in cardiovascular disease expenditures in Europe (average costs 630 EUR/person/year) (14, 30).

Therefore, accurately differentiating between real AMIs and MINOCAs is essential for effective resource allocation and management of costs.

The true costs of treatment and management for patients with MINOCA with regard to high care monitoring, such as in an ICU, are currently unknown and can only be approximated based on the known costs of ICU patients with underlying cardiovascular diseases.

In the context of cost considerations, our research shows a variability within the data, indicating that there may be outliers or a high degree of heterogeneity within the MINOCA and MICAD patient population. In our analysis, this variability can be attributed to differences in patient demographics, comorbidities, and the severity of conditions within the MINOCA and MICAD groups that required different length of stay in hospital and in a high care unit expressed through varying health care cost expenditures. The variability in costs and resource utilization underscores the need for personalized approaches in managing MINOCA and MICAD patients, as standardized protocols may not adequately address the diverse needs of the patient population. It is important to recognize that our cost analysis is based on healthcare resource data specific to Germany, and may not be fully applicable to other countries, including those within Europe. Nevertheless, factors such as in-hospital treatment, duration of high care monitoring or total length of stay offer valuable insights into resource utilization and are comparable for different health care systems.

Whether a patient with MINOCA should receive high care monitoring or a normal admission may depend on the severity of their condition and their overall prognosis. Some patients with MINOCA may be able to receive care on a normal hospital ward, while others may require more intensive care or a specialized cardiac unit. A non-existing in-hospital and significantly lower 30-day mortality in the MINOCA group after the clinical index event speak for a generally more favorable course compared to “true infarctions.”

Given limited personnel and financial resources and the obligation to be economically viable, it would be advisable to establish an effective treatment path at the first contact with the patient, which occurs in over 70% via the emergency department, in order to make organizational and administrative processes as efficient as possible. One approach could be the consistent application of the 1 h algorithm recommended by the ESC (31). A faster “rule-in” allows infarction patients to be treated earlier, avoiding long-term complications and mortality due to delayed diagnosis; and ultimately reduces resource consumption and costs (31).

The early use of computed tomography (CT) to rule out CAD might allow a step-down approach, e.g., early discharge or admission to a regular ward for further diagnostic work-up for patients who generally carry a low to intermediate risk, and would avoid an overuse of high care monitoring capacities (32, 33).

Another way to diagnose MINOCA is through cardiac MRI or invasive coronary imaging and vascular function tests such as IVUS, OCT or pressure wire (2, 17, 18). These methods provide additional information that can help differentiate MINOCA from other types of heart disease (2, 17, 18). It is important to note that these diagnostic tests should not be performed in an outpatient setting. Rather, they require specialized expertise and infrastructure available at a hospital or specialized center.

However, further research is needed to better understand different causes of MINOCA, and ultimately formulate recommendations for risk stratification and management of MINOCA.

## Limitations

This is a single-center and retrospective study with associated limitations and missing data in some cases. A potential bias cannot be excluded. The study was conducted at a large university heart center in Germany. Therefore, economic analysis and derived implications can only be formulated for the German health care system.

The presence of wide standard deviations suggests that while our mean estimates provide a central tendency of expenditure for MINOCA, there is a substantial variability that needs to be considered when interpreting the results. Clinicians should be aware of the variability of the costs and consider individual patient characteristics and contextual factors when applying our study findings.

Other aspects that should be considered as limitations but can be the focus of future studies include: the rate of readmissions/ medication compliance /medication rate/quality of life of MICAD vs. MINOCA patients.

## Conclusion

Despite MINOCA requiring an almost exclusively conservative treatment it utilizes relevant financial and medical resources including experienced personal, an interdisciplinary team, high care monitoring and an in-hospital treatment of relevant length. Regarding scarcity of structural and personnel resources, this entity should be put under particular consideration for refining care concepts and identification of patients suitable for early discharge and outpatient care.

## Data Availability

The datasets presented in this article are not readily available because the data presented in this study are not publicly available due to local legal restrictions on data safety. Requests to access the datasets should be directed to Franz.Haertel@med.uni-jena.de.
